# No association between variation in the *NR4A1 *gene locus and metabolic traits in white subjects at increased risk for type 2 diabetes

**DOI:** 10.1186/1471-2350-11-84

**Published:** 2010-06-04

**Authors:** Karsten Müssig, Fausto Machicao, Jürgen Machann, Fritz Schick, Claus D Claussen, Norbert Stefan, Andreas Fritsche, Hans-Ulrich Häring, Harald Staiger

**Affiliations:** 1 Division of Endocrinology, Diabetology, Angiology, Nephrology, and Clinical Chemistry, Department of Internal Medicine, University Hospital of Tübingen, Otfried-Müller-Str. 10, 72076 Tübingen, Germany; 2 Section on Experimental Radiology, Department of Diagnostic Radiology, University Hospital of Tübingen, Hoppe-Seyler-Str. 3, 72076 Tübingen, Germany; 3 Division of Nutritional and Preventive Medicine, Department of Internal Medicine, University Hospital of Tübingen, Otfried-Müller-Str. 10, 72076 Tübingen, Germany

## Abstract

**Background:**

The nuclear receptor NR4A1 is implicated in metabolic regulation in insulin-sensitive tissues, such as liver, adipose tissue, and skeletal muscle. Functional loss of NR4A1 results in insulin resistance and enhanced intramuscular and hepatic lipid content. Therefore, we investigated in a cohort of white European subjects at increased risk for type 2 diabetes whether genetic variation within the *NR4A1 *gene locus contributes to prediabetic phenotypes, such as insulin resistance, ectopic fat distribution, or β-cell dysfunction.

**Methods:**

We genotyped 1495 subjects (989 women, 506 men) for five single nucleotide polymorphisms (SNPs) tagging 100% of common variants (MAF = 0.05) within the *NR4A1 *gene locus with an r^2 ^= 0.8. All subjects underwent an oral glucose tolerance test (OGTT), a subset additionally had a hyperinsulinemic-euglycemic clamp (n = 506). Ectopic hepatic (n = 296) and intramyocellular (n = 264) lipids were determined by magnetic resonance spectroscopy. Peak aerobic capacity, a surrogate parameter for oxidative capacity of skeletal muscle, was measured by an incremental exercise test on a motorized treadmill (n = 270).

**Results:**

After appropriate adjustment and Bonferroni correction for multiple comparisons, none of the five SNPs was reliably associated with insulin sensitivity, ectopic fat distribution, peak aerobic capacity, or indices of insulin secretion (all p ≥ 0.05).

**Conclusions:**

Our data suggest that common genetic variation within the *NR4A1 *gene locus may not play a major role in the development of prediabetic phenotypes in our white European population.

## Background

In addition to peripheral insulin resistance and pancreatic beta-cell dysfunction, type 2 diabetes mellitus is also characterized by aberrant hepatic gluconeogenesis. cAMP response element-binding protein (CREB), a key regulator of hepatic gluconeogenesis, mediates its actions through transcriptional induction of the nuclear hormone receptor coactivator PGC-1α (peroxisome proliferator-activated (PPAR)-γ coactivator-1α). Recently, CREB-induced activation of the NR4A orphan nuclear receptor family, including the three highly homologous isotypes, NR4A1, NR4A2, and NR4A3 (also known as Nur77, Nurr1, and Nor1), has been identified as a novel PGC-1α-independent mechanism for regulating hepatic gluconeogenesis [1]. The same nuclear receptors are also implicated in metabolic regulation in other insulin-sensitive tissues. NR4A1 inhibits adipocyte differentiation and regulates expression of genes linked to glucose metabolism in skeletal muscle [2,3]. In a very recent study, functional loss of NR4A1 was reported to result in exacerbated insulin resistance in both skeletal muscle and liver and to increase intramuscular and hepatic lipid content upon high-fat diet [4].

In light of these data, *NR4A1 *appears to be an attractive prediabetes candidate gene. Therefore, we studied the impact of common genetic variation within the *NR4A1 *gene locus on prediabetes phenotypes, including insulin resistance, ectopic fat distribution, and, as we have recently found an association between common polymorphisms within the *NR4A3 *locus and insulin release [5], also β-cell dysfunction.

## Methods

### Subjects

The 1495 non-diabetic white subjects at increased risk of type 2 diabetes mellitus were recruited from the southern part of Germany and participated in an ongoing study on the pathophysiology of type 2 diabetes [6]. All subjects were metabolically characterized by an oral glucose tolerance test (OGTT). In randomly selected subgroups, a hyperinsulinemic-euglycemic clamp was performed, intramyocellular lipids (IMCL) and intrahepatic lipids were determined by magnetic resonance spectroscopy (MRS), peak aerobic capacity, a surrogate parameter for oxidative capacity of skeletal muscle, was measured using an incremental exercise test on a motorized treadmill (Saturn; HP-Cosmos, Traunstein, Germany) [6]. Participants gave informed written consent to the study. The protocol was approved by the local ethical committee.

### Genotyping

Using the publically available phase II data of the International HapMap Project derived from a population of Utah residents with ancestry from northern and western Europe (release #24 November 2008, http://www.hapmap.org/index.html.en), we screened in silico the complete *NR4A1 *gene locus spanning 15,798 bases from nucleotide 50,723,763 to nucleotide 50,739,552 (7 exons, located on human chromosome 12q13) as well as 5 kb of its 5'-flanking region and 3 kb of its 3'-flanking regions. Among thirteen informative single nucleotide polymorphisms (SNPs), the five SNPs rs2242107 C/T, rs1283155 C/T, rs744690 T/G, rs2603751 A/G (all located in non-coding regions of the gene locus), and rs2701124 C/T (located in the coding region resulting in a synonymous substitution) were chosen (Additional File 1), covering 100% of common variants (minor allele frequency [MAF] = 0.05) within the *NR4A1 *gene with an r^2 ^= 0.8, according to Tagger analysis http://www.broad.mit.edu/mpg/tagger. Genotyping was performed using the TaqMan assay (Applied Biosystems, Foster City, CA). The overall genotyping success rate was 99.8% (all SNPs 100%, except for rs1283155: 99.1%), and rescreening of 3% of subjects gave 100% identical results. Genotypes were verified in a random sample of 50 subjects by bidirectional sequencing.

### Statistical analyses

In order to approximate normal distribution, log_*e*_-transformation of the following metabolic variables was performed prior to simple and multivariate linear regression analyses: body mass index, waist circumference, fasting glucose, glucose at 120 min. during OGTT, homeostasis model assessment of insulin resistance (HOMA-IR), OGTT- and clamp-derived insulin sensitivity index (ISI), the ratio of area under the curve (AUC) insulin to AUC glucose at 30 min. during OGTT, the ratio of AUC C-peptide to AUC glucose during OGTT, insulinogenic index, hepatic lipids, intramyocellular lipids (IMCL) in tibialis anterior and soleus muscles, and peak aerobic capacity. In multivariate linear regression models, the trait was chosen as dependent variable, and gender, age, body mass index (BMI), and genotype were tested as independent variables. To account for the number of SNPs analysed (n = 5), a Bonferroni-corrected α-level of p < 0.01 was considered statistically significant. Bonferroni correction was not performed for the number of traits given that the traits were interrelated. The statistical software package JMP 7.0 (SAS Institue, Cary, NC) was used. Hardy-Weinberg equilibrium was tested using the χ^2 ^test. The effect sizes detectable in the different cohorts undergoing an OGTT, a hyperinsulinemic-euglycemic clamp, and MRS were ≥ 8%, ≥ 14%, and ≥ 18% in the additive model and ≥ 20%, ≥ 36%, and ≥ 43% in the dominant model, respectively. Power calculation was performed in the additive inheritance model by F-tests and in the dominant inheritance model by two-tailed t-tests (1-β>0.8) using G*power software available at http://www.psycho.uni-duesseldorf.de/aap/projects/gpower/.

## Results

### Characterization and genotyping of the study population

Characteristics of the 1495 genotyped non-diabetic subjects (989 women, 506 men) from the southwest of Germany are shown in Additional File 2. The five *NR4A1 *SNPs were in Hardy-Weinberg equilibrium (all p > 0.5). The observed and the HapMap genotype distributions as well as linkage disequilibrium (LD) statistics are shown in Additional File 1 and Additional File 3, respectively.

### Associations between *NR4A1* SNPs and metabolic traits

After appropriate adjustment and Bonferroni correction for multiple comparisons, the five *NR4A1 *SNPs were not significantly associated with insulin sensitivity, indices of insulin secretion, ectopic fat distribution, or peak aerobic capacity (Tables 1 and 2, Additional Files 4 and 5), except for an association between rs1283155 and glucose at 120 min. of the OGTT in the additive inheritance model (p = 0.0078). However, in the dominant model, this association was no longer significant (p = 0.0153). Furthermore, no allele dose effect was seen with this association.

**Table 1:** Associations of *NR4A1 *SNPs rs744690, rs2603751, and rs2242107 with metabolic parameters (n = 1495).

*SNP*	*rs744690*					*rs2603751*					*rs2242107*				
**Genotype**	**TT**	**TG**	**GG**	**P**_add_.	**P**_dom_.	**AA**	**AG**	**GG**	**P**_add_.	**P**_dom_.	**CC**	**CT**	**TT**	**P**_add_.	**P**_dom_.
N	1066	384	39	-	-	1156	313	20	-	-	769	589	131	-	-
BMI (kg/m^2^)	28.4 ± 8.1	28.4 ± 7.5	28.3 ± 7.4	0.8	0.6	28.5 ± 7.9	28.8 ± 8.2	29.6 ± 11.1	0.8	0.5	28.6 ± 7.9	28.4 ± 7.9	29.2 ± 8.8	0.7	0.9
Waist circumference (cm)	93 ± 17	94 ± 17	92 ± 15	0.7	0.9	94 ± 17	94 ± 18	91 ± 20	0.9	0.9	94 ± 17	93 ± 17	95 ± 18	0.4	0.4
Glucose, fasting (mM)	5.09 ± 0.55	5.10 ± 0.55	5.15 ± 0.54	0.8	0.5	5.10 ± 0.55	5.07 ± 0.54	4.96 ± 0.53	0.3	0.2	5.11 ± 0.56	5.07 ± 0.54	5.12 ± 0.53	0.7	0.4
Glucose, 120 min. OGTT (mM)	6.28 ± 1.65	6.11 ± 1.67	6.47 ± 1.73	0.14	0.08	6.24 ± 1.67	6.25 ± 1.63	6.09 ± 1.46	0.9	1.0	6.29 ± 1.68	6.17 ± 1.62	6.25 ± 1.68	0.4	0.15
HOMA-IR (U)	2.44 ± 2.11	2.36 ± 2.20	2.47 ± 2.73	0.8	1.0	2.42 ± 2.16	2.40 ± 2.12	2.78 ± 2.33	0.5	0.4	2.49 ± 2.13	2.28 ± 2.07	2.65 ± 2.57	0.06	0.06
ISI, OGTT (U)	16.7 ± 11.1	16.6 ± 10.3	15.4 ± 7.7	0.9	0.7	16.6 ± 10.9	16.6 ± 10.7	15.4 ± 11.1	0.7	0.7	16.4 ± 11.0	17.1 ± 10.8	15.7 ± 9.8	0.13	0.08
ISI, clamp (U)^#^	0.086 ± 0.053	0.086 ± 0.061	0.081 ± 0.040	0.9	0.6	0.086 ± 0.054	0.088 ± 0.058	0.046 ± 0.013	0.3	0.4	0.086 ± 0.054	0.085 ± 0.054	0.085 ± 0.064	0.7	0.8
AUC Ins [30 min.]/AUC glc [30 min.] (pM/mM)	40.5 ± 29.7	40.8 ± 32.0	35.2 ± 18.2	0.6	0.5	40.4 ± 29.1	40.0 ± 33.4	47.5 ± 29.4	0.2	0.3	41.0 ± 30.2	38.8 ± 27.5	44.1 ± 39.0	0.8	0.9
AUC C-pep/AUC glc (pM/mM)	317 ± 104	324 ± 117	304 ± 82	0.7	0.5	317 ± 106	324 ± 111	325 ± 111	0.6	0.3	318 ± 105	317 ± 106	331 ± 121	0.5	0.4
Insulinogenic index (pM/mM)	50.5 ± 40.5	51.8 ± 43.5	42.2 ± 22.8	0.7	0.4	50.6 ± 39.7	49.9 ± 45.4	58.6 ± 36.0	0.4	0.5	51.2 ± 41.4	48.7 ± 37.1	55.7 ± 52.6	0.6	0.8

Raw data are presented and given as means ± SD. For statistical analysis, data were log_*e*_-transformed. AUC Insulin [30 min.]/AUC glc [30 min.], AUC C-pep/AUC glc, and insulinogenic index were adjusted for gender, age, BMI, and ISI-OGTT. Fasting glucose, glucose at 120 min. of the OGTT, HOMA-IR, ISI-OGTT, and ISI-clamp were adjusted for gender, age, and BMI. BMI and waist circumference were adjusted for gender and age. AUC - area under the curve; BMI - body mass index; C-pep - C-peptide; glc - glucose; HOMA-IR - homeostasis model assessment of insulin resistance; ISI - insulin sensitivity index; OGTT - oral glucose tolerance test; p_>add_. - p-value in the addidtive inheritance model; p_dom_. - p-value in the dominant inheritance model; SNP - single nucleotide polymorphism; U - units. ^#^N = 506.

Insulinogenic index was assessed as the ratio of (insulin at 30 min. of the OGTT - fasting insulin) to glucose at 30 min. of the OGTT.

**Table 2:** Associations of *NR4A1 *SNPs rs2701124 and rs1283155 with metabolic parameters (n = 1495).

*SNP*	*rs2701124*					*rs1283155*				
**Genotype**	**CC**	**CT**	**TT**	**P**_add_.	**P**_dom_.	**CC**	**CT**	**TT**	**P**_add_.	**P**_dom_.
N	1242	239	8	-	-	885	507	83	-	-
BMI (kg/m^2^)	28.5 ± 7.8	29.0 ± 8.6	32.3 ± 16.1	0.5	0.3	28.7 ± 8.2	28.5 ± 7.7	28.3 ± 7.7	0.8	0.6
Waist circumference (cm)	94 ± 17	93 ± 19	93 ± 28	1.0	0.8	94 ± 18	94 ± 17	93 ± 16	0.9	0.8
Glucose, fasting (mM)	5.10 ± 0.55	5.06 ± 0.54	5.11 ± 0.64	0.6	0.3	5.10 ± 0.55	5.10 ± 0.56	5.04 ± 0.48	0.4	0.7
Glucose, 120 min. OGTT (mM)	6.25 ± 1.66	6.22 ± 1.64	5.94 ± 1.75	0.7	0.7	6.32 ± 1.67	6.07 ± 1.62	6.46 ± 1.66	0.0078	0.0153
HOMA-IR (U)	2.41 ± 2.11	2.47 ± 2.35	2.68 ± 1.82	0.5	0.2	2.51 ± 2.30	2.31 ± 1.87	2.25 ± 2.28	0.3	0.17
ISI, OGTT (U)	16.6 ± 10.9	16.7 ± 10.5	16.1 ± 13.2	0.7	0.4	16.3 ± 10.7	16.9 ± 10.9	17.1 ± 10.9	0.2	0.08
ISI, clamp (U)^#^	0.086 ± 0.054	0.087 ± 0.058	0.054 ± 0.001	0.8	0.5	0.084 ± 0.052	0.089 ± 0.061	0.090 ± 0.046	0.7	0.9
AUC Insulin [30 min.]/AUC glc [30 min.] (pM/mM)	40.2 ± 29.0	41.4 ± 35.5	44.9 ± 23.8	0.9	0.6	41.3 ± 30.8	39.7 ± 28.4	37.8 ± 34.0	0.7	0.8
AUC C-pep/AUC glc (pM/mM)	317 ± 106	329 ± 113	312 ± 72	0.2	0.10	320 ± 103	320 ± 113	304 ± 108	0.6	0.9
Insulinogenic index (pM/mM)	50.3 ± 39.4	52.1 ± 48.4	56.1 ± 32.8	0.9	0.7	51.5 ± 41.7	50.0 ± 39.3	46.7 ± 44.6	0.8	0.6

Raw data are presented and given as means ± SD. For statistical analysis, data were log_*e*_-transformed. AUC Insulin [30 min.]/AUC glc [30 min.], AUC C-pep/AUC glc, and insulinogenic index were adjusted for gender, age, BMI, and ISI-OGTT. Fasting glucose, glucose at 120 min. of the OGTT, HOMA-IR, ISI-OGTT, and ISI-clamp were adjusted for gender, age, and BMI. BMI and waist circumference were adjusted for gender and age. AUC - area under the curve; BMI - body mass index; C-pep - C-peptide; glc - glucose; HOMA-IR - homeostasis model assessment of insulin resistance; ISI - insulin sensitivity index; OGTT - oral glucose tolerance test; p_add_. - p-value in the addidtive inheritance model; p_dom_. - p-value in the dominant inheritance model; SNP - single nucleotide polymorphism; U - units. ^# ^ISI (clamp) data were available from 506 subjects.

Insulinogenic index was assessed as the ratio of (insulin at 30 min. of the OGTT - fasting insulin) to glucose at 30 min. of the OGTT.

## Discussion

Genotyping of a metabolically well-characterized population for *NR4A1 *SNPs revealed no reliable association of this gene locus with insulin sensitivity, insulin secretion, or ectopic fat distribution. For some traits, e.g., IMCL or liver fat content, our study was sufficiently powered to detect only moderate effect sizes. Therefore, the lack of association between *NR4A1 *gene variants and ectopic fat distribution has to be ultimately ruled out in larger studies with comparable measurements, such as magnetic resonance imaging (MRI) or computed tomography (CT). In line with this, recent genome-wide association studies showed that large cohorts are required to detect small effect sizes of diabetic traits, such as fasting glucose and insulin [7]. Furthermore, given that only SNPs with a MAF greater than 5% were chosen, we cannot exclude that rarer variants may be associated with prediabetic phenotypes.

## Conclusions

In conclusion, our data suggest that common variation within the *NR4A1 *gene locus may not play a major role in the development of prediabetic phenotypes, such as insulin resistance, β-cell dysfunction, or disproportionate fat distribution, in our white European population at an increased risk for type 2 diabetes.

## Abbreviations

BMI: body mass index; CREB: cAMP response element-binding protein; CT: computed tomography; IMCL: intramyocellular lipids; LD: linkage disequilibrium; MAF: minor allele frequency; MRI: magnetic resonance imaging; MRS: magnetic resonance spectroscopy; OGTT: oral glucose tolerance test; PGC-1α: peroxisome proliferator-activated (PPAR)-γ coactivator-1α; SNP: single nucleotide polymorphisms.

## Competing interests

The authors declare that they have no competing interests.

## Authors' contributions

KM prepared all tables and figures and wrote the first draft of the manuscript. KM and HS were responsible for the complete statistical analysis. HS revised the manuscript. FM is responsible of the genotyping facility at the University of Tübingen. JM, FS, and CDC performed the magnetic resonance spectroscopy investigation. NS, AF and HUH acted as principal investigators for the study, and were responsible for patient and data management. All authors read and approved the final manuscript.

## Pre-publication history

The pre-publication history for this paper can be accessed here:

http://www.biomedcentral.com/1471-2350/11/84/prepub

## Supplementary Material

Additional file 1 **Genomic region of human chromosome 12 harbouring the *NR4A1 *gene locus and linkage disequilibrium (LD) data of representative SNPs within this region (HapMap data).** The *NR4A1 *gene consists of 7 exons and spans 15,798 bases from nucleotide 50,723,763 to nucleotide 50,739,552. The locations of the genotyped representative SNPs are indicated by arrows. The HapMap minor allele frequencies (MAF) are given below the SNP numbers. The Haploview LD colour scheme 'r-squared' was chosen to visualize LD data. Within the diamonds, the r^2 ^values are given. Figure. Click here for file

Additional file 2 **Clinical characteristics of the overall cohort, the hyperinsulinemic-euglycemic clamp subgroup, and the magnetic resonance spectroscopy subgroup**. Table. Click here for file

Additional file 3 **Observed linkage disequilibrium statistics (D', r^2^) among the five representative *NR4A1 *SNPs covering 100% of the common genetic variation (MAF = minor allele frequency)**. Table. Click here for file

Additional file 4 **Associations of *NR4A1 *SNPs rs744690, rs2603751, and rs2242107 with ectopic lipids and muscle respiratory capacity (n = 301)**. Table. Click here for file

Additional file 5 **Associations of *NR4A1 *SNPs rs2701124 and rs1283155 with ectopic lipids and muscle respiratory capacity (n = 301)**. Table. Click here for file
