# Supplementary figures and images for: No association between variation in the NR4A1 gene locus and metabolic traits in white subjects at increased risk for type 2 diabetes
Source: BMC Med Genet. 2010 Jun 4;11:84. doi: 10.1186/1471-2350-11-84 (PMC2894787; doi:10.1186/1471-2350-11-84)

## Slide 1
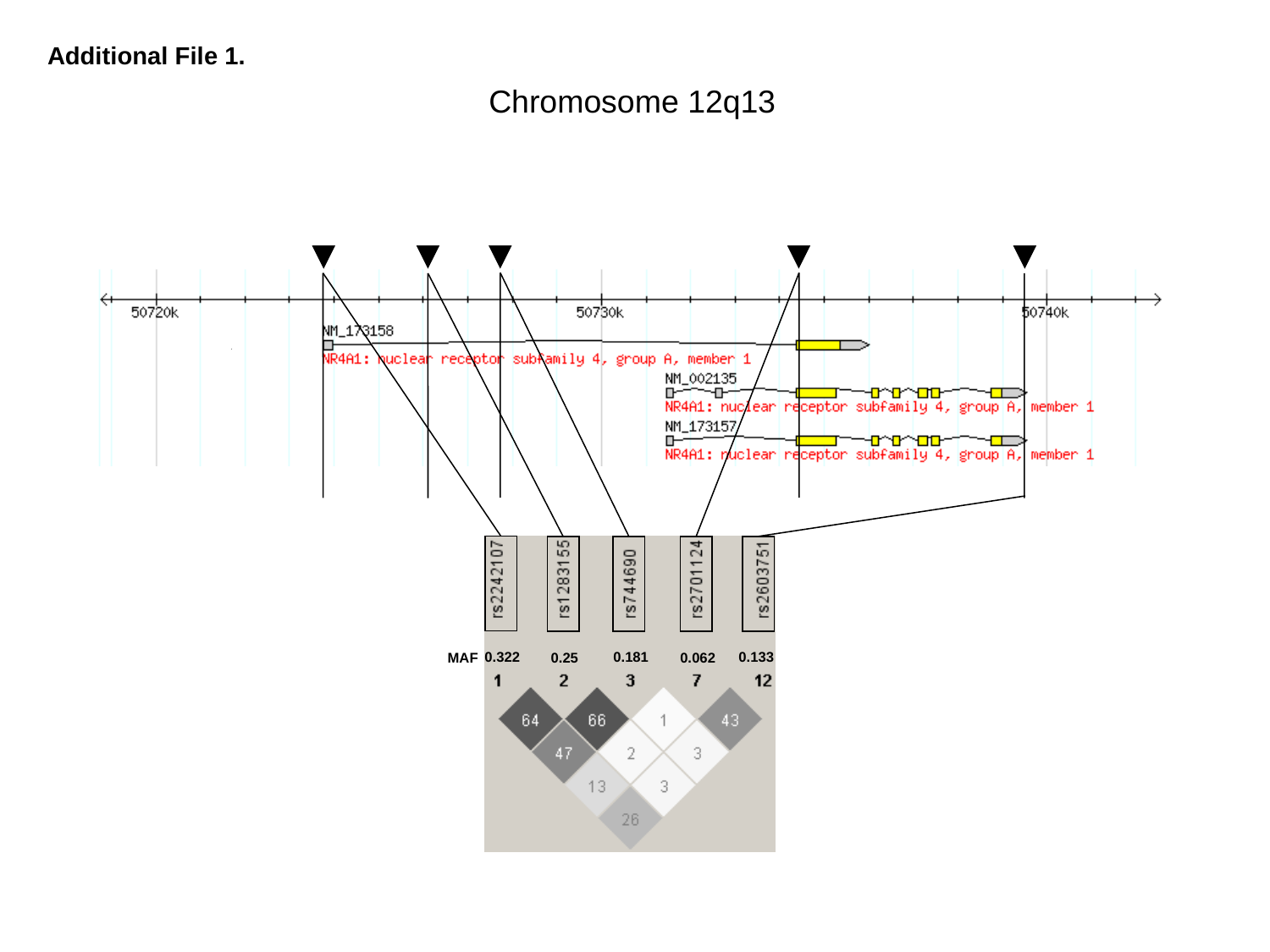

Additional File 1.
Chromosome 12q13
0.322
0.181
0.133
MAF
0.25
0.062

Supplement: Additional file 1 — Genomic region of human chromosome 12 harbouring the NR4A1 gene locus and linkage disequilibrium (LD) data of representative SNPs within this region (HapMap data). The NR4A1 gene consists of 7 exons and spans 15,798 bases from nucleotide 50,723,763 to nucleotide 50,739,552. The locations of the genotyped representative SNPs are indicated by arrows. The HapMap minor allele frequencies (MAF) are given below the SNP numbers. The Haploview LD colour scheme 'r-squared' was chosen to visualize LD data. Within the diamonds, the r2 values are given. Figure. [file 1471-2350-11-84-S1.PPT]
