# Supplementary material for: No association between variation in the NR4A1 gene locus and metabolic traits in white subjects at increased risk for type 2 diabetes
Source: BMC Med Genet. 2010 Jun 4;11:84. doi: 10.1186/1471-2350-11-84 (PMC2894787; doi:10.1186/1471-2350-11-84)
Supplement: Additional file 2 — Clinical characteristics of the overall cohort, the hyperinsulinemic-euglycemic clamp subgroup, and the magnetic resonance spectroscopy subgroup. Table. [file 1471-2350-11-84-S2.DOC]

**Additional File 2.** Clinical characteristics of the overall cohort, the hyperinsulinemic-euglycemic clamp subgroup, and the magnetic resonance spectroscopy subgroup.

|  | Overall cohort  (N=1495) | Clamp subgroup  (N=506) | MRS subgroup  (N=301) |
| --- | --- | --- | --- |
| Gender (male / female) | 506 / 989 | 235 / 271 | 120 / 181 |
| IGT / IFG / (IGT + IFG) | 142 / 150 / 113 | 46 / 38 / 30 | 38 / 34 / 33 |
| Age (y) | 39±13 | 39±12 | 45±12 |
| BMI (kg/m^2^) | 28.6±8.0 | 27.1±5.7 | 29.4±4.8 |
| Waist circumference (cm) | 94±17 | 92±15 | 97±13 |
| Fasting glucose (mM) | 5.10±0.55 | 5.00±0.54 | 5.22±0.49 |
| Glucose 120 min. OGTT (mM) | 6.24±1.66 | 6.12±1.71 | 6.88±1.57 |
| Fasting insulin (pM) | 62.4±51.2 | 52.1±35.8 | 60.2±35.8 |
| Insulin 30 min. OGTT (pM) | 481±384 | 435±318 | 515±353 |

Data are presented as means±SD. BMI, body mass index; IGT, impaired glucose tolerance; IFG, impaired fasting glucose; MRS, magnetic resonance spectroscopy; OGTT, oral glucose tolerance test.
