# Supplementary material for: No association between variation in the NR4A1 gene locus and metabolic traits in white subjects at increased risk for type 2 diabetes
Source: BMC Med Genet. 2010 Jun 4;11:84. doi: 10.1186/1471-2350-11-84 (PMC2894787; doi:10.1186/1471-2350-11-84)
Supplement: Additional file 3 — Observed linkage disequilibrium statistics (D', r2) among the five representative NR4A1 SNPs covering 100% of the common genetic variation (MAF = minor allele frequency). Table. [file 1471-2350-11-84-S3.DOC]

**Additional File 3.** Observed linkage disequilibrium statistics (D’, r²) among the five representative SNPs covering 100 % of the common genetic variation (MAF = minor allele frequency).

| SNP | rs2242107 | rs1283155 | rs744690 | rs2701124 | rs2603751 |
| --- | --- | --- | --- | --- | --- |
| rs2242107 | - | **0.728** | **0.962** | **0.803** | **0.780** |
| rs1283155 | 0.392 | - | **0.964** | **0.465** | **0.110** |
| rs744690 | 0.426 | 0.581 | - | **0.803** | **0.842** |
| rs2701124 | 0.151 | 0.006 | 0.011 | - | **0.982** |
| rs2603751 | 0.204 | 0.005 | 0.018 | 0.672 | - |
| **MAF** | 0.286 | 0.229 | 0.156 | 0.086 | 0.118 |

Bold: D’ values; non-bold: r² values.
