# Supplementary material for: No association between variation in the NR4A1 gene locus and metabolic traits in white subjects at increased risk for type 2 diabetes
Source: BMC Med Genet. 2010 Jun 4;11:84. doi: 10.1186/1471-2350-11-84 (PMC2894787; doi:10.1186/1471-2350-11-84)
Supplement: Additional file 4 — Associations of NR4A1 SNPs rs744690, rs2603751, and rs2242107 with ectopic lipids and muscle respiratory capacity (n = 301). Table. [file 1471-2350-11-84-S4.DOC]

**Additional File 4.** Associations of *NR4A1* SNPs rs744690, rs2603751, and rs2242107 with ectopic lipids and muscle respiratory capacity (n=301).

| SNP | rs744690 | | |  | | rs2603751 | | |  | | rs2242107 | | |  | |
| --- | --- | --- | --- | --- | --- | --- | --- | --- | --- | --- | --- | --- | --- | --- | --- |
| Genotype | TT | TG | GG | P_add_. | P_dom_. | AA | AG | GG | P_add_. | P_dom_. | CC | CT | TT | P_add_. | P_dom_. |
| N | 215 | 75 | 11 | - | - | 230 | 67 | 4 | - | - | 153 | 123 | 25 | - | - |
| BMI (kg/m^2^) | 29.4 ±4.8 | 29.7 ±5.0 | 27.1 ±3.2 | 0.3 | 1.0 | 29.2 ±4.4 | 29.6 ±6.0 | 32.0 ±2.2 | 0.4 | 0.6 | 29.5 ±4.5 | 29.3 ±5.3 | 28.5 ±4.2 | 0.6 | 0.5 |
| Waist circumference (cm) | 96 ±13 | 98 ±13 | 93 ±13 | 0.09 | 0.6 | 96 ±12 | 97 ±16 | 100 ±8 | 0.6 | 1.0 | 97 ±12 | 96 ±14 | 97 ±13 | 0.6 | 0.4 |
| Hepatic lipids (%) ^#^ | 5.5 ±5.9 | 5.6 ±6.2 | 5.8 ±6.3 | 0.9 | 0.7 | 5.5 ±6.0 | 5.7 ±5.8 | 6.8 ±4.3 | 0.8 | 0.9 | 5.8 ±6.0 | 4.9 ±5.6 | 6.8 ±7.0 | 0.2 | 0.14 |
| IMCL tibialis anterior (AU) ^§^ | 4.1 ±1.9 | 3.7 ±1.7 | 3.5 ±0.9 | 0.3 | 0.16 | 4.0 ±1.9 | 4.1 ±1.6 | 3.0 ±1.5 | 0.2 | 0.7 | 4.1 ±1.9 | 4.0 ±1.8 | 3.5 ±1.2 | 0.6 | 0.4 |
| IMCL soleus (AU) | 15.5 ±7.7 | 15.1 ±7.7 | 13.1 ±5.8 | 0.7 | 0.5 | 14.8 ±6.5 | 17.4 ±10.8 | 12.6 ±4.5 | 0.6 | 0.7 | 15.1 ±6.4 | 15.4 ±8.7 | 16.8 ±9.6 | 1.0 | 0.9 |
| VO_2 peak TM_ (ml·min^–1^·kg lbm^–1^) ^$^ | 24.7 ±6.6 | 24.5 ±6.5 | 23.4 ±6.8 | 0.4 | 0.9 | 24.6 ±6.1 | 24.9 ±7.9 | 19.7 ±1.1 | 0.8 | 1.0 | 24.6 ±5.9 | 25.0 ±7.4 | 22.4 ±5.4 | 0.04 | 1.0 |

Raw data are presented and given as means ±SD. For statistical analysis, data were log-transformed. BMI and waist circumference were adjusted for gender and age. All other parameters were adjusted for gender, age, and BMI. BMI – body mass index; IMCL – intramyocellular lipids; lbm – lean body mass; p_add_. – p-value in the addidtive inheritance model; p_dom_. – p-value in the dominant inheritance model; SNP – single nucleotide polymorphism; U – units; VO_2 peak_ - peak aerobic capacity. ^#^ N=296, ^§^ N=264, ^$^N=270.
