# Supplementary material for: No association between variation in the NR4A1 gene locus and metabolic traits in white subjects at increased risk for type 2 diabetes
Source: BMC Med Genet. 2010 Jun 4;11:84. doi: 10.1186/1471-2350-11-84 (PMC2894787; doi:10.1186/1471-2350-11-84)
Supplement: Additional file 5 — Associations of NR4A1 SNPs rs2701124 and rs1283155 with ectopic lipids and muscle respiratory capacity (n = 301). Table. [file 1471-2350-11-84-S5.DOC]

**Additional File 5.** Associations of *NR4A1* SNPs rs2701124 and rs1283155 with ectopic lipids and muscle respiratory capacity (n=301).

| SNP | rs2701124 | | |  | | rs1283155 | | |  | |
| --- | --- | --- | --- | --- | --- | --- | --- | --- | --- | --- |
| Genotype | CC | CT | TT | P_add_. | P_dom_. | CC | CT | TT | P_add_. | P_dom_. |
| N | 253 | 46 | 2 | - | - | 180 | 100 | 20 | - | - |
| BMI (kg/m^2^) | 29.3 ±4.5 | 29.8 ±6.1 | 32.2 ±3.2 | 0.6 | 0.6 | 29.4 ±4.8 | 29.5 ±4.9 | 28.0 ±4.5 | 0.5 | 0.8 |
| Waist circumference (cm) | 97 ±13 | 97 ±16 | 96 ±1 | 0.7 | 0.7 | 96 ±13 | 97 ±14 | 94 ±12 | 0.4 | 0.9 |
| Hepatic lipids (%) ^#^ | 5.4 ±6.0 | 6.4 ±5.9 | 3.5 ±2.9 | 0.4 | 0.2 | 5.4 ±5.8 | 5.9 ±6.5 | 5.3 ±5.2 | 0.9 | 0.8 |
| IMCL tibialis anterior (AU) ^§^ | 4.0 ±1.9 | 4.3 ±1.5 | 3.2 ±1.6 | 0.4 | 0.3 | 4.2 ±2.0 | 3.7 ±1.6 | 3.5 ±1.2 | 0.15 | 0.05 |
| IMCL soleus (AU) | 15.1 ±7.6 | 16.7 ±8.0 | 10.3 ±3.1 | 0.4 | 0.7 | 15.1 ±6.5 | 16.1 ±9.6 | 13.0 ±5.4 | 0.5 | 0.9 |
| VO_2 peak TM_ (ml·min^–1^·kg lbm^–1^) ^$^ | 24.5 ±6.2 | 25.1 ±8.2 | 19.0 ±1.2 | 0.9 | 1.0 | 24.5 ±6.5 | 24.9 ±6.5 | 23.1 ±6.6 | 0.4 | 0.8 |

Raw data are presented and given as means ±SD. For statistical analysis, data were log-transformed. BMI and waist circumference were adjusted for gender and age. All other parameters were adjusted for gender, age, and BMI. BMI – body mass index; IMCL – intramyocellular lipids; lbm – lean body mass; p_add_. – p-value in the addidtive inheritance model; p_dom_. – p-value in the dominant inheritance model; SNP – single nucleotide polymorphism; U – units; VO_2 peak_ - peak aerobic capacity. ^#^ N=296, ^§^ N=264, ^$^N=270.
